# Clinical features, pathophysiological mechanisms, and multidisciplinary management strategies for rhinitis-induced adenoid facies in children and adolescents: a review

**DOI:** 10.3389/falgy.2025.1650119

**Published:** 2025-09-02

**Authors:** Ying Ding, Yan Xu, Shanshan Han, Min Gao, Long Wang, Shanshan Xu, Ting Guo, Huiwen Bai

**Affiliations:** 1 Pediatric Hospital of the First Affiliated Hospital of Henan University of Chinese Medicine, Zhengzhou, China; 2 Pediatric College of Henan University of Chinese Medicine, Zhengzhou, China

**Keywords:** rhinitis face, adenoid facies, mouth breathing, children, adolescents, maxillofacial development, allergic rhinitis, multidisciplinary management

## Abstract

Chronic rhinitis and its associated persistent nasal obstruction and mouth breathing are core factors leading to the development of characteristic “rhinitis face” or “adenoid facies” in children and adolescents. This review elucidates the diverse clinical manifestations of “rhinitis face,” including: persistent open-mouth posture; abnormal patterns of facial skeletal growth, such as midface hypoplasia and increased lower anterior facial height resulting in “long face syndrome”; alterations in jaw morphology and position, including maxillary constriction, high-arched palate, and mandibular retrognathia or posterior-inferior rotation; and various dentoalveolar malocclusions, such as proclined maxillary incisors, lip incompetence, narrow dental arches, and open bite. Additionally, these include characteristic periorbital skin changes, such as “allergic shiners” (dark circles under the eyes due to venous stasis or pigmentation), Dennie-Morgan lines (infraorbital folds associated with atopy), and, in some patients, eyelash trichomegaly (increased eyelash growth) potentially due to chronic inflammation. The nose may also exhibit a transverse nasal crease (the “allergic salute” sign) from repetitive rubbing. This paper delves into its pathophysiological mechanisms, emphasizing that mouth breathing patterns triggered by chronic nasal airway obstruction are the initiating factor. This alters the equilibrium of orofacial muscle forces, interferes with normal tongue posture and function, and affects the normal growth trajectory of the maxillofacial skeleton. Combined with local inflammatory responses and mechanical stimuli, these factors collectively contribute to the development of these complex facial characteristics. Clinical assessment requires a comprehensive approach including medical history, detailed physical examination, and various ancillary investigations such as nasal endoscopy, imaging studies (x-ray, CT, CBCT), cephalometric analysis, nasal patency tests, and allergen testing. “Rhinitis face” not only affects aesthetics but can also lead to severe maxillofacial skeletal deformities, dental malocclusions, temporomandibular joint dysfunction, and sleep-disordered breathing. It can also profoundly impact respiratory physiology, exercise tolerance, speech clarity, psychological well-being, and quality of life. Its long-term effects can persist into adulthood, although skeletal adaptive changes diminish after growth cessation. Regarding gender differences in its prevalence, existing data suggest that upstream factors (such as obstructive sleep apnea) may have a higher prevalence in males, and the impact of mouth breathing on facial morphology might exhibit sex-specific differences. However, the overall sex ratio for “rhinitis face” remains inconclusive. Concerning the notion that rhinitis causes enlarged eyes, there is currently no scientific evidence to support an actual increase in eyeball size. The perception of “larger eyes” is more likely a visual contrast effect due to allergic shiners, Dennie-Morgan lines, and possible mild eyelid edema. Regarding public opinions about finding “rhinitis face in girls” attractive, this review emphasizes the lack of scientific basis for such views, which are more likely subjective perceptions or cultural phenomena. Medically, “rhinitis face” is considered a pathological condition requiring active intervention. Management strategies for affected children emphasize a multidisciplinary approach, including early diagnosis and active treatment of the primary nasal pathology (e.g., allergic rhinitis, adenoidal hypertrophy), correction of improper mouth breathing habits through methods like orofacial myofunctional therapy, and, when necessary, intervention by orthodontists or maxillofacial surgeons (e.g., rapid maxillary expansion, fixed orthodontic treatment). This review aims to provide clinicians with a comprehensive understanding of “rhinitis face” to facilitate its early recognition, standardized diagnosis and treatment, and comprehensive management.

## 1 Introduction

Chronic rhinitis, particularly allergic rhinitis, is an extremely common inflammatory respiratory disease globally, significantly and negatively impacting patients’ quality of life (1). Persistent rhinitis not only causes typical respiratory symptoms such as nasal obstruction, rhinorrhea, sneezing, and nasal itching (1, 2), but may also lead to a series of unique, recognizable changes in facial features. These changes are colloquially known in clinical practice as “rhinitis face” or “allergic facies,” and these facial characteristics are often more prominent and typical, especially when patients concurrently have significant adenoidal hypertrophy (3). Childhood is a period of high incidence for rhinitis. Persistent nasal symptoms, particularly chronic nasal obstruction, are often secondary to underlying conditions such as allergic rhinitis, chronic rhinosinusitis, or adenoidal hypertrophy. This long-term, difficult-to-alleviate nasal airway obstruction often compels affected children to abandon physiological nasal breathing and adopt a compensatory pattern of mouth breathing (4, 5). This abnormal mouth breathing pattern is widely considered a key initiating factor and central element in the development of the so-called “rhinitis face” or “adenoid facies,” capable of exerting profound and complex adverse effects on the normal growth trajectory of facial bones, the functional equilibrium of the orofacial musculature, and normal dental alignment and occlusion (3).

In this review, we refer to the syndrome of characteristic facial alterations driven by chronic rhinitis as “rhinitic facies.” It should be clarified that this clinical entity is largely synonymous in its clinical presentation with the traditionally described “adenoid facies.” However, the term “rhinitic facies” is used throughout this paper to consistently emphasize “chronic rhinitis” as the core upstream etiology and initiating factor. It is a comprehensive clinical term for a series of characteristic changes in the maxillofacial and related anatomical regions caused by persistent nasal obstruction and secondary mouth breathing, which result from long-term chronic nasal inflammation (such as allergic rhinitis) and associated pathological states (adenoid hypertrophy) (3, 6). These features collectively constitute the distinctive physical appearance of affected children and primarily include: persistent open-mouth posture (4); alterations in facial skeletal morphology, such as a long, narrow face, excessive vertical growth of the lower third of the face (forming the so-called “long face syndrome”) (6, 7), potentially underdeveloped maxilla, high-arched palate, steepened palatal plane (8), and often posteriorly and inferiorly rotated mandible leading to a retrognathic appearance (7, 8); intraoral changes, such as various dental misalignments and malocclusions (including proclined maxillary incisors, lip incompetence, narrow dental arches, posterior crossbite, and open bite) (8, 9), and alterations in lip features (e.g., short or everted upper lip, flaccid and thickened lower lip, incomplete lip seal) (10); ocular features, including “allergic shiners” (11), Dennie-Morgan lines (11–13), potential eyelash trichomegaly in some patients (14, 15) (the appearance of “larger” eyes being more a visual effect than actual enlargement); nasal features, such as the “allergic salute” sign (transverse nasal crease) and underdeveloped alar cartilages; and other possible manifestations like listlessness, poor concentration, and low tongue posture (16).

These facial changes, induced by rhinitis and subsequent mouth breathing, warrant significant attention from clinicians and parents, particularly during the peak growth and development periods of children and adolescents. This is because they not only directly affect aesthetics but may also herald potential, more severe maxillofacial developmental anomalies, sleep-disordered breathing, and a range of long-term health problems. Therefore, early and accurate identification and assessment of “rhinitis face” and its associated etiological factors, followed by effective, multidisciplinary interventions based on these assessments, are of crucial clinical value and public health significance for promoting the healthy growth of children and adolescents and significantly improving their current and future quality of life.

The objectives of this review are, through a comprehensive and systematic review and synthesis of existing national and international medical literature, to: 1. clearly define the clinical concept and scope of “rhinitis face” and describe its diverse clinical manifestations in detail; 2. thoroughly explore its pathophysiological mechanisms, particularly the core role and cascade effects of chronic nasal obstruction and mouth breathing; 3. systematically summarize current objective clinical methods and ancillary diagnostic tools used to assess these facial changes; 4. comprehensively elucidate the multifaceted, long-term clinical impacts and potential complications associated with “rhinitis face”; and 5. outline and evaluate current multidisciplinary intervention strategies and comprehensive management plans for “rhinitis face.”

The clinical significance and anticipated value of this review are primarily manifested as follows: Firstly, to provide clinicians in relevant fields such as otolaryngology, dentistry (especially orthodontics), pediatrics, allergology, and general practice with an up-to-date, comprehensive, and in-depth summary of knowledge regarding “rhinitis face.” This aims to enhance their ability for early recognition of this syndrome, diagnostic accuracy, and the level of standardized, individualized treatment. Secondly, by scientifically clarifying common misconceptions (such as those regarding “aesthetic preferences” or it causing “enlarged eyes”), to help improve public understanding of the true health nature of “rhinitis face,” and to guide patients and their families towards seeking timely medical attention and scientific management. Finally, by reviewing the progress and limitations of existing research, to offer valuable references and inspiration for future in-depth clinical and basic research in this field, ultimately serving to improve the health and well-being of the patient population affected by this condition.

## 2 Etiology and pathophysiological mechanisms of “rhinitis face”

The development of “rhinitis face” is the result of an interplay among multiple factors and pathophysiological processes, with the core element being persistent nasal airway obstruction due to chronic nasal inflammation and the consequent compensatory mouth breathing.

### 2.1 Chronic nasal inflammation and the core role of nasal obstruction

Allergic rhinitis (AR) is one of the most common causes of chronic nasal obstruction in children (1, 17). In AR, recurrent stimulation by allergens [e.g., pet dander (1), pollen (2), dust mites (18)] triggers an IgE-mediated inflammatory response in the nasal mucosa, leading to nasal mucosal edema, increased vascular permeability, and increased mucus secretion, thereby causing nasal obstruction (1, 2, 19). Chronic non-allergic rhinitis and chronic rhinosinusitis (CRS), sometimes with nasal polyps, are also important causes of long-term nasal obstruction (20). Adenoidal hypertrophy (AH), common in childhood, is another major factor causing nasopharyngeal obstruction, leading to nasal obstruction and mouth breathing, with a prevalence in children as high as 49.7% (3). AH itself may be caused by factors such as recurrent infections, inflammation, or allergies. Severe AH significantly reduces nasopharyngeal airway volume and increases airflow resistance (21). A persistent inflammatory state, such as the action of cytokines like IL-8, maintains and exacerbates nasal obstruction symptoms (20).

### 2.2 Mouth breathing and its cascading impact on maxillofacial development

Prolonged nasal obstruction compels patients, especially children and adolescents in their growth and development period, to adopt a compensatory habit of mouth breathing (4, 16). Normal nasal breathing, by generating physiological air pressure and flow, is crucial for maintaining the balance of the orofacial muscular system and guiding the normal growth and development of the maxillofacial skeleton. When the breathing pattern shifts to mouth breathing, a series of chain reactions ensues.

Firstly, abnormal tongue posture is a key alteration. During normal nasal breathing, the tongue primarily contacts the palate, providing essential support and physiological stimulation for the transverse and anteroposterior development of the maxilla. However, in a mouth breathing pattern, to maintain oral airway patency, the tongue is often forced into a low position on the floor of the mouth or protrudes anteriorly and inferiorly (16). This abnormal tongue posture deprives the maxilla of its normal physiological stimulation and support, potentially leading to insufficient transverse development of the maxillary dental arch (i.e., maxillary constriction) and an abnormally high-arched palate over time (8).

Secondly, perioral muscle imbalance follows. During mouth breathing, the tone of the orbicularis oris muscle, which maintains lip seal, is typically weakened, leading to lip incompetence, where the lips cannot close naturally at rest. Concurrently, the buccinator muscles in the cheeks may exhibit relatively increased tone or abnormal contraction due to resisting abnormal airflow or participating in compensatory respiratory movements; this imbalanced muscular force further exacerbates the tendency towards maxillary arch constriction.

Furthermore, the position and growth direction of the mandible are altered. To enlarge the oropharyngeal airway space, mouth breathers often adopt a mandible position characterized by posterior and inferior (clockwise) rotation. This long-term postural change leads to a significant increase in the lower anterior facial height, an increased gonial angle (angle between the mandibular ramus and body), and a steepened mandibular plane angle (angle formed by the lower border of the mandible with the anterior cranial base or Frankfort horizontal plane), collectively forming the characteristic high-angle facial type, often described clinically as “adenoid facies” or “long face syndrome” (7, 8, 22). A study on children and adolescents clearly demonstrated that, compared to nasal breathers, mouth breathers exhibited greater palatal length, increased lower anterior facial height, and a lower hyoid bone position, all objective indicators of the impact of mouth breathing on craniofacial structures (16).

Moreover, the specific effects of these changes on the jaws and teeth are significant. Animal experiments have confirmed that during the adolescent growth period, even unilateral nasal obstruction can hinder the normal length development of the ipsilateral mandible and the growth of the nasomaxillary complex length (23). More in-depth animal studies revealed that persistent mouth breathing can lead to pathological changes in the mandibular condylar cartilage (articular growth center) of adolescent rats, including reduced cartilage thickness, subchondral bone resorption, decreased expression of key cartilage matrix components (such as type II collagen and aggrecan), and can induce chondrocyte apoptosis (a process potentially mediated jointly by extrinsic and mitochondrial apoptotic pathways) (24). Intermittent bilateral nasal obstruction in adolescent rat models also led to developmental defects of the mandibular condyle, possibly related to its impact on the normal differentiation capacity of mesenchymal stem cells in the condylar region into chondrocytes (25). Clinical observations are consistent with these findings; children who mouth breathe often exhibit an increased facial convexity angle, while female mouth breathers may additionally present with insufficient transverse mandibular width development (4). A retrospective study comparing dentofacial characteristics of mouth breathers across different age groups showed that: in childhood (typically 5–12 years), mouth breathing was significantly associated with Class II malocclusion (i.e., mandible positioned posteriorly relative to the maxilla, presenting as mandibular retrognathia), mandibular hypoplasia (short mandible), and a retrognathic skeletal pattern; in adolescence (typically 13–18 years), mouth breathing remained associated with Class II malocclusion and short mandible, and was also characterized by increased lower anterior facial height; upon reaching adulthood, the direct association between mouth breathing and skeletal factors diminished, with changes more often manifesting as dental compensations to adapt to the abnormal skeletal base (26). These dentofacial changes can specifically manifest as proclination or protrusion of maxillary incisors, increased (deep bite) or decreased (open bite) overbite, posterior crossbite, and dental crowding, among other malocclusions (8, 9).

Finally, given these cascading negative impacts induced by mouth breathing, the importance of early intervention is self-evident. Intervention during the peak growth and development period in children, for instance, through orthodontic treatments such as Rapid Maxillary Expansion (RME), is often employed to actively address mouth breathing habits and associated maxillofacial developmental abnormalities, aiming to halt or mitigate the further progression of these adverse effects (27, 28).

### 2.3 Mechanisms of local skin changes

The local skin changes involved in “rhinitis face,” such as allergic shiners, Dennie-Morgan lines, and the transverse nasal crease, have distinct formation mechanisms, although all are closely related to chronic nasal inflammation and its secondary behaviors.

Regarding the formation of **Allergic shiners**, the primary pathophysiological basis lies in the chronic inflammatory state of the nasal and paranasal sinus regions. This persistent inflammation leads to local microvascular dilation and increased vascular wall permeability. The skin of the lower eyelid is one of the thinnest areas of the human body, with loose subcutaneous tissue and a rich, superficial capillary network. When chronic rhino-sinusitis is present, it can cause impaired venous return or stasis in the periorbital area, particularly the lower eyelid region. This venous stasis deepens the color of the blood visible through the thin skin, giving the infraorbital skin its characteristic bluish-purple or dusky appearance, i.e., clinical “dark circles” (11). Furthermore, rhinitis patients often experience ocular pruritus; repetitive eye rubbing can also cause minor damage to local capillaries and post-inflammatory hyperpigmentation, further exacerbating the appearance of dark circles.

The formation of Dennie-Morgan lines (DMF), i.e., skin folds beneath the lower eyelids, is considered the result of multiple interacting factors. Firstly, DMF are closely associated with chronic eczematous dermatitis changes in the lower eyelid. Chronic inflammation of the lower eyelid, whether due to atopic dermatitis (AD) or allergic contact dermatitis, can lead to local skin thickening, edema, dryness, and reduced elasticity, thereby promoting skin fold formation (13). One study found that in AD patients with lower eyelid dermatitis, the incidence of DMF could be as high as 83% (13). Secondly, repetitive mechanical irritation is an important factor. For instance, due to allergy-related pruritus (especially the intense nocturnal pruritus common in AD patients), patients habitually rub their eyelids upward or outward. This long-term physical friction may lead to chronic skin damage, disorganization of elastic fibers, and the formation of permanent creases (12). Some scholars speculate that sleep fragmentation caused by nocturnal pruritus may interfere with the normal rhythmic secretion of melatonin, thereby affecting microcirculation in the infraorbital skin, leading to increased blood perfusion and tissue fluid exudation. This chronic edematous state may exacerbate collagen fiber dysregulation, thus promoting the formation and deepening of DMF (12). Furthermore, DMF are also widely considered a clinical marker of a broader “atopic diathesis.” They are not only very common in AD patients but also have a significantly higher incidence in patients without obvious skin eczema but with other atopic respiratory diseases (such as allergic rhinitis, asthma) compared to control groups with non-atopic diseases (11). This strongly suggests that an individual's genetic susceptibility, congenital defects in skin barrier function, or specific response patterns to inflammatory stimuli may play important roles in the formation of DMF.

As for the Nasal crease/Allergic salute, its formation mechanism is more direct. This results from patients (especially children) involuntarily and habitually rubbing the tip of their nose upward and outward or sideways with the palm base or fingers to alleviate nasal itching symptoms caused by rhinitis and to attempt to improve nasal airflow. This signature gesture is known as the “allergic salute.” Prolonged and repetitive friction and upward pushing forces on the skin of the lower third of the nasal bridge eventually form one or more transverse hyperpigmented or superficial skin creases perpendicular to the direction of frown lines, i.e., the nasal crease.

### 2.4 Discussion of “long eyelashes” and “large eyes” phenomena

In clinical practice, it is indeed observed that some children suffering from long-term rhinitis appear to have longer and thicker eyelashes and seemingly larger eyes than their peers. The mechanisms behind these phenomena warrant discussion.

Regarding long eyelashes (Eyelash trichomegaly), existing medical literature provides some support for this phenomenon. A systematic review clearly indicates that both allergic rhinitis and atopic dermatitis are listed as acquired etiologies associated with excessive eyelash growth (i.e., eyelash trichomegaly) (14). Another well-designed case-control study further found that children with atopic dermatitis (AD) had significantly longer eyelashes than healthy control children. More importantly, this study also found that AD disease severity (assessed by SCORAD score >50 representing moderate to severe), concurrent palmar hyperlinearity (deepened and increased palm lines), and significantly elevated serum total IgE levels were all significantly associated with abnormal eyelash growth. This series of findings suggests that markedly long eyelashes might serve as an indirect, easily observable clinical surrogate marker for assessing the severity of childhood AD (15). Although the phenomenon exists, its precise biological mechanisms are not yet fully understood. It is speculated to be related to the following factors: 1. chronic inflammatory state: A long-term chronic inflammatory microenvironment in the periorbital region (including hair follicles at the eyelid margin) might release certain cytokines or growth factors that directly or indirectly stimulate the anagen phase of hair follicles, prolonging hair growth time, thus leading to longer and thicker eyelashes. 2. Endocrine or immunomodulatory changes associated with atopic diathesis: Atopic individuals may have specific growth factor profiles or immune response patterns; these intrinsic factors might systematically promote hair growth. However, these hypotheses require further experimental research for confirmation.

Regarding the so-called “large eyes” phenomenon, there is currently a lack of direct and reliable scientific evidence to suggest that chronic rhinitis or its related conditions (such as allergic conjunctivitis) can cause an actual, organic enlargement of the eyeball itself (axial length or orbital volume). The clinically observed impression of “large eyes” is more likely a visual relative effect or illusion resulting from a combination of multiple factors. Possible reasons include: a. presence of Allergic shiners: As mentioned earlier, the contrast between the dark infraorbital skin and the surrounding normal skin tone visually enhances the orbital contour, making the eyes themselves appear more prominent and distinct within the face, thereby giving the impression of “larger” eyes. b. Influence of Dennie-Morgan lines: Skin folds formed in the lower eyelid area may, to some extent, alter the local anatomical appearance and light-shadow relationships around the eyes, indirectly causing visual changes. c. Mild eyelid edema: During recurrent episodes of chronic allergic conjunctivitis or rhinitis, mild, chronic edema of the eyelid tissues may occur, especially during acute exacerbations or upon waking. Although usually not severe, this edema can be sufficient to make the eyes appear slightly “puffy” or “prominent,” thus visually creating the impression of “larger” eyes. These speculative explanations for the “large eyes” phenomenon also require further clinical research based on objective measurements and imaging assessments for confirmation or revision.

## 3 Clinical assessment and diagnosis of “rhinitis face”

### 3.1 Recognition of clinical signs

The diagnosis of “rhinitis face” primarily relies on detailed history taking and comprehensive clinical physical examination. During history taking, emphasis should be placed on the type of rhinitis (e.g., allergic, non-allergic), duration of illness, seasonal characteristics of symptoms, known or suspected triggers (e.g., allergen exposure, climate changes), severity of major symptoms [e.g., using a Visual Analog Scale (VAS) to assess the distress caused by nasal obstruction, rhinorrhea, sneezing, nasal pruritus, ocular pruritus, pharyngeal pruritus], and the presence of sleep-related issues such as habitual snoring, mouth breathing, restless sleep, or even apnea.

During physical examination, clinicians should carefully and systematically observe whether the patient exhibits the typical facial signs detailed in Section 2.2 above. This includes assessing for persistent open-mouth posture and lip incompetence; observing facial type characteristics, such as a long, narrow face (tendency towards long face syndrome) and mandibular retrognathia (micrognathic appearance); examining lip morphology, such as a short upper lip or flaccid, thickened lower lip; and evaluating dental alignment for malocclusions like protrusion, crowding, crossbite, or open bite. Concurrently, special attention must be paid to the periorbital skin for the presence of allergic shiners and Dennie-Morgan lines, and to the nose for the typical transverse crease (allergic salute). The S2k guideline issued by the German Society of Oto-Rhino-Laryngology, Head and Neck Surgery also specifically emphasizes that when examining children with suspected adenoidal hypertrophy, routine attention should be paid to the clinical manifestations of “adenoid facies” (i.e., long face syndrome), typically characterized by chronic mouth breathing, sometimes with the tongue tip habitually positioned between the lips or protruding outwards (6). In addition to meticulous observation of facial features, routine ENT examination is indispensable, requiring assessment of the intranasal condition (including color and swelling of the nasal mucosa, nature and amount of secretions, size of bilateral inferior turbinates, presence of significant septal deviation, and any new growths such as nasal polyps in the nasal passages) and the oropharyngeal condition (such as tonsillar symmetry and grading, and morphology of the palatal arches).

### 3.2 Ancillary diagnostic methods

A range of ancillary diagnostic methods is often employed clinically for a more comprehensive and objective assessment of “rhinitis face” and its underlying causes.

1. Specialized ENT Examinations play a core role in this process. Nasal endoscopy provides high-definition, magnified images of intranasal structures, allowing direct observation and assessment of the degree of mucosal inflammation (e.g., hyperemia, edema, pallor), the actual size of turbinates and their degree of airway obstruction, the nature and origin of nasal secretions, septal morphology (presence of deviation or spurs), and, crucially, the size of nasopharyngeal adenoids and their degree of choanal obstruction (29). Furthermore, acoustic rhinometry and computed rhinomanometry are two non-invasive nasal function tests. The former measures serial nasal cavity cross-sectional areas using sound wave reflection, while the latter directly measures airflow through the nasal cavity and transnasal pressure difference to calculate nasal resistance. Both methods can objectively and quantitatively assess the minimum effective ventilation cross-sectional area of the nasal cavity and actual nasal airflow resistance, providing an objective basis for judging the severity of nasal obstruction and its impact on respiratory function (30).
2. Imaging Assessment is crucial for revealing skeletal structures and soft tissue pathologies. Traditional lateral neck radiographs were widely used to assess adenoid size [e.g., by measuring the adenoid-nasopharyngeal (A/N) ratio or using the Fujioka grading method], but their accuracy is susceptible to various factors such as projection posture, exposure conditions, and the subjective experience of the reader. Recently, artificial intelligence (AI)-based automated diagnostic methods have been proposed to improve the objectivity and accuracy of adenoidal hypertrophy diagnosis (31). In contrast, computed tomography (CT) or cone-beam CT (CBCT) can more precisely display the complex three-dimensional anatomical structures of the nasal cavity, paranasal sinuses, and nasopharynx, as well as the extent of possible lesions (e.g., nasal polyps, sinusitis). CBCT, in particular, can provide clear 3D volumetric information of the upper airway region at a relatively low radiation dose, offering superior value over traditional 2D imaging techniques for assessing the true extent of nasopharyngeal airway stenosis and its impact on surrounding structures (32). CT scans can also be used for precise measurement of the coronal width of the nasal cavity and maxilla, providing reference for orthodontic treatment (30). Cephalometric analysis, whether based on 2D radiographs or 3D CBCT reconstructed images, is one of the “gold standard” methods for assessing craniofacial skeletal morphology, dento-osseous relationships, anteroposterior airway dimensions, and soft tissue profile. By precisely measuring a series of preset craniofacial landmarks, planes, and angles (e.g., SNA angle, SNB angle, ANB angle, GoGn-SN angle, lower facial height, posterior airway space width), cephalometric analysis can objectively quantify skeletal developmental abnormalities associated with “rhinitis face,” such as maxillary development status (protrusion or retrusion), mandibular position and rotation direction, and dysregulation of vertical facial height proportions (7, 8, 26, 33, 34).
3. Respiratory Function and Physiological Assessment helps understand the impact of nasal obstruction and mouth breathing on the overall respiratory system. Impulse oscillometry (IOS) is a non-invasive lung function test particularly suitable for children due to its simple operation and lack of need for special patient cooperation. IOS can assess total airway resistance and differentiate between upper and lower airway resistance and reactance components, aiding in determining the specific impact of mouth breathing on respiratory mechanics (29). Polysomnography (PSG) is the gold standard for diagnosing sleep-disordered breathing (SDB) (such as obstructive sleep apnea-hypopnea syndrome, OSAHS). It comprehensively and continuously monitors multiple physiological parameters during sleep, including electroencephalogram (EEG), electrooculogram (EOG), electromyogram (EMG), electrocardiogram (ECG), respiratory airflow, thoracoabdominal movements, blood oxygen saturation, and body position, thereby accurately assessing sleep architecture, type and frequency of respiratory events, and severity of nocturnal hypoxia (35). The 6-minute walk test (6MWT) is a simple submaximal exercise test that indirectly assesses cardiopulmonary reserve and overall exercise tolerance by measuring the maximum distance a patient can walk briskly on a flat surface in 6 min. Studies have used it to compare physical activity capacity between mouth-breathing and nasal-breathing children (36).
4. Allergen Testing is a key step in diagnosing allergic rhinitis. For patients with rhinitis suspected to be caused by allergic factors, skin prick tests (SPT) or serum specific IgE (sIgE) antibody tests should be routinely performed. These tests help identify the specific allergens (e.g., dust mites, pollen, molds, animal dander) to which the patient is sensitized, providing an important basis for subsequent allergen avoidance guidance and selection of specific immunotherapy regimens (1).
5. Orofacial Myofunctional Assessment has also gained increasing attention in recent years. 3D facial scanning technology can rapidly, non-invasively, and precisely acquire and quantify three-dimensional morphological data of facial soft tissues using optical scanning principles, such as facial convexity angle, mandibular width, and lower lip height parameters. This provides a new, objective tool for clinically assessing the impact of mouth breathing on soft tissue appearance and monitoring treatment effects (4) Maxillofacial surface electromyography (sEMG) can non-invasively record the electrical activity patterns of specific perioral muscles (such as orbicularis oris, mentalis) at rest and during specific functional activities (e.g., swallowing, chewing, phonation) via electrodes placed on their surface. Studies have shown that mouth-breathing patients often exhibit abnormal perioral muscle activity patterns. Nomogram models based on sEMG data have been developed to aid in diagnosing mouth breathing, contributing to earlier, more objective identification of this behavior and supporting timely intervention (5).
6. Application of Clinical Questionnaires and Scales is also an important component of the assessment system. Various validated standardized questionnaires can be used from the patient's or parent's perspective to assess the severity of rhinitis symptoms [e.g., VAS, Total Nasal Symptom Score (TNSS)/Total Ocular Symptom Score (TOSS), Sino-Nasal Outcome Test-22 (SNOT-22)], the impact of the disease on quality of life [e.g., Rhinoconjunctivitis Quality of Life Questionnaire (RQLQ), Pediatric Rhinoconjunctivitis Quality of Life Questionnaire (PRQLQ)], and sleep-related problems [e.g., Pediatric Sleep Questionnaire (PSQ), Obstructive Sleep Apnea-18 (OSA-18) quality of life survey] (35). These questionnaires provide valuable references for quantifying subjective feelings and monitoring treatment efficacy.

## 4 Clinical impact and consequences of “rhinitis face”

### 4.1 Long-term effects on maxillofacial structure and function

Prolonged mouth breathing habits and associated orofacial muscle dysfunction can cause significant, sometimes irreversible, long-term effects on the maxillofacial structures of children and adolescents during critical growth and development periods. These effects may persist or evolve even after skeletal growth has largely ceased in adulthood, due to soft tissue compensation or ongoing pathophysiological processes, although skeletal adaptive changes themselves diminish after growth completion (26). These long-term effects are primarily manifested in the following aspects:

Firstly, in terms of dental alignment and arch form, mouth breathing is a significant risk factor for various malocclusions and dental arch dysmorphologies. Clinically common malocclusions associated with mouth breathing include Class II malocclusion (i.e., mandible positioned posteriorly relative to the maxilla, presenting as mandibular retrognathia), proclination or protrusion of maxillary incisors, anterior open bite (especially in the anterior region, where upper and lower incisors do not contact during occlusion), deep or shallow overbite, insufficient transverse development of dental arches (particularly maxillary arch constriction, resulting in a “V-shaped” or narrow “U-shaped” arch), posterior crossbite (i.e., mandibular posterior teeth occlude buccal to maxillary posterior teeth), and dental crowding due to insufficient arch length (8, 9, 26). Among these, maxillary arch constriction and a high-arched palate (Gothic palate) are particularly common and characteristic features in mouth-breathing patients (8).

Secondly, regarding the overall facial profile, mouth breathing can lead to typical facial changes, known as “adenoid facies” or “long face syndrome.” Key features include excessive vertical development of facial height, especially a significant increase in lower anterior facial height (from the nasal base to the menton). Concurrently, the mandible often exhibits a posterior and inferior rotational tendency, leading to increased mandibular plane angle (angle between the lower border of the mandible and the anterior cranial base) and gonial angle (angle between the posterior border of the mandibular ramus and the lower border of the mandibular body). The mandibular body length may be relatively insufficient or appear retrognathic due to its posterior-inferior rotation. Soft tissue features often include everted lips, lip incompetence (i.e., inability to achieve complete lip seal at rest, with tooth exposure), and alae nasi that may appear relatively collapsed or underdeveloped due to chronic mouth breathing and nasal disuse (7, 8, 22). A study using 3D facial scanning technology clearly indicated that mouth breathing was significantly associated with an increased facial convexity angle in school-aged male children, while in female children, it was associated with restricted transverse mandibular width development and increased lower lip height (4). Other research has shown that severe adenoidal hypertrophy in childhood can lead to a significantly retropositioned upper and lower lip relative to facial esthetic reference lines (such as the Ricketts E-line, connecting the tip of the nose to the pogonion), affecting facial profile harmony (10).

Furthermore, long-term mouth breathing may affect temporomandibular joint (TMJ) function, inducing or exacerbating temporomandibular joint dysfunction (TMD). Mouth breathing has been identified as a risk factor for TMD-related signs and symptoms in children and adolescents (such as pain in the joint area or masticatory muscles, joint clicking or crepitus, limited mouth opening, or abnormal mandibular movement patterns) (37). This may be related to the abnormal mandibular position, malocclusion, and consequent masticatory muscle dysfunction and overload caused by mouth breathing.

Additionally, mouth breathing may influence hyoid bone position and head posture. Studies have observed an abnormally low hyoid bone position in mouth-breathing adolescents (16). The hyoid bone is an important structure in the oropharyngeal airway, and its positional changes may be related to compensatory postural adjustments made by the body to maintain oropharyngeal airway patency. Some studies also suggest that long-term mouth breathing patterns may further affect head posture by influencing the tension balance of cervical muscles, for instance, leading to the development of a forward head posture; however, evidence for the association between mouth breathing and head posture is currently inconsistent and requires further research for confirmation.

Finally, animal experimental studies have revealed potential adverse effects of mouth breathing on condylar (mandibular condyle) development. Results indicate that simulated mouth breathing (or direct nasal obstruction) can lead to thinning of the mandibular condylar cartilage, subchondral bone resorption, reduced synthesis of cartilage matrix components (such as type II collagen and aggrecan), and can induce chondrocyte apoptosis in growing adolescent rats. These pathological changes undoubtedly affect normal condylar growth and remodeling and may form a potential structural basis for future TMD development (24, 25).

### 4.2 Impact on respiratory physiology and sleep quality

Chronic nasal obstruction leading to long-term mouth breathing not only affects maxillofacial structures but also has a significant negative impact on an individual's respiratory physiology and sleep quality.

Firstly, mouth breathing leads to reduced respiratory efficiency and increased airway resistance. The nasal cavity, the initial part of the upper respiratory tract, has important physiological functions of warming, humidifying, and filtering inhaled air. When individuals chronically adopt mouth breathing, these vital nasal physiological functions are bypassed, and large volumes of inadequately processed air (dry, cold, and potentially containing more dust and pathogens) directly irritate the pharyngeal and lower airway mucosa. This not only easily induces or exacerbates airway inflammation but may also reduce respiratory efficiency. Studies have shown that children who mouth breathe may exhibit impaired respiratory function early on. For example, impulse oscillometry (IOS) findings indicate that, compared to nasal-breathing children, mouth-breathing children typically have higher total airway resistance (Rtot), suggesting reduced overall airflow capacity. Their upper and lower airway reactance (Xrs) parameters may also show abnormalities, reflecting changes in airway elastic properties (29).

Secondly, mouth breathing may lead to decreased exercise tolerance. Effective gas exchange is fundamental to maintaining physical activity capacity. The mouth breathing pattern, possibly by failing to fully utilize the physiological regulatory functions of the nasal cavity or due to its association with abnormal respiratory mechanics, can affect gas exchange efficiency and oxygen utilization during exercise. This causes individuals to fatigue more easily during physical activity, with a consequent decline in exercise tolerance. Studies using the 6-minute walk test (6MWT), a simple method for assessing exercise tolerance, have shown that mouth-breathing children and adolescents may cover shorter distances in the 6MWT than their nasal-breathing peers, and their relevant cardiorespiratory parameters (such as heart rate and blood oxygen saturation changes) during the test may also be inferior (36).

Thirdly, and one of the most clinically significant impacts, is the close association between mouth breathing and sleep-disordered breathing (SDB). Chronic nasal obstruction and resultant nocturnal mouth breathing are major causes or important aggravating factors for the development and progression of pediatric obstructive sleep apnea-hypopnea syndrome (OSAHS). Persistent nocturnal mouth breathing and insufficient nasal ventilation readily lead to frequent snoring, apnea (complete cessation of airflow), or hypopnea (significant reduction in airflow) events. These respiratory events further disrupt normal sleep architecture [e.g., reducing deep sleep and rapid eye movement (REM) sleep proportions] and can lead to intermittent nocturnal hypoxemia. Long-term SDB not only severely affects a child's daytime alertness (e.g., somnolence, poor concentration), learning ability, and behavior (e.g., hyperactivity, irritability) but, in severe and persistent cases, may also impact normal growth and development due to factors such as suppressed growth hormone secretion (35).

### 4.3 Impact on psychological well-being and quality of life

The conspicuous external features associated with “rhinitis face,” such as persistent skin changes (e.g., allergic shiners, Dennie-Morgan lines), striking facial dysmorphology (e.g., long face, mandibular retrognathia, lip incompetence), and potentially very obvious dental malocclusions, can all have a non-negligible negative impact on the patient's psychological well-being and overall quality of life.

Firstly, regarding negative emotions and self-perception, patients may experience a range of psychological distress. Atopic dermatitis (AD), a chronic inflammatory skin disease that often coexists with allergic rhinitis and can also manifest with facial features like Dennie-Morgan lines, serves as an example. Studies on AD patients show they often experience strong feelings of uncertainty and frustration when symptoms worsen. More importantly, due to the chronic persistence of the disease and often unsatisfactory treatment outcomes, patients may gradually adapt to or underestimate the actual negative impact of the disease on their quality of life; therefore, providing continuous psychological and emotional support is crucial for patients with such chronic conditions (38). Similar psychological states and needs could very well arise in patients who feel their image is damaged and self-esteem is undermined by the appearance changes caused by “rhinitis face.” They may consequently feel inferior, anxious, and even exhibit social avoidance behaviors.

Secondly, “rhinitis face” can also present numerous challenges in social and family contexts. For children and adolescents whose “rhinitis face” has led to obvious dental malocclusions or other facial abnormalities, their distinctive appearance may make them an object of peer attention, discussion, or even ridicule, undoubtedly negatively affecting the development of their self-confidence and normal social interactions. A study on 11–14-year-old children with malocclusion and their mothers found that, compared to the children themselves, mothers typically expressed stronger dissatisfaction with their child's dental appearance and also tended to overestimate the degree of negative impact of malocclusion on their child's emotional health (39). This finding suggests that the distress caused by “rhinitis face” is not limited to the patient alone but may also impose additional psychological stress, worry, and anxiety on their family members (especially parents).

Finally, considering the overall impact on quality of life, chronic rhinitis symptoms themselves (e.g., persistent nasal obstruction, rhinorrhea, headache), resultant sleep disturbances (e.g., snoring, choking, nocturnal awakenings), unavoidable daytime fatigue, potential decline in academic performance or work efficiency, and persistent anxiety and dissatisfaction with their own appearance—these factors collectively pose a severe challenge to the patient's overall quality of life, potentially impairing them on multiple levels, including physical, psychological, and social functioning (1).

### 4.4 Impact on speech and articulation

Chronic nasal obstruction, long-term mouth breathing, and the resultant series of orofacial structural abnormalities (such as dental malalignment, high-arched palate, abnormal tongue posture) can have multifaceted adverse effects on an individual's speech and articulation functions, manifested as follows:

Firstly, abnormalities in resonance function may occur. The most common is hyponasality, also known as rhinolalia clausa. When the nasal cavity or nasopharynx is significantly obstructed due to severe rhinitis, severe adenoidal hypertrophy, or other causes, airflow cannot pass normally through the nasal cavity to participate in resonance. This causes nasal consonants (e.g., /m/, /n/, and /ŋ/ in English) to sound muffled and lack normal nasal resonance, similar to the stuffy nasal sound typical of a “cold” or “speaking as if with a blocked nose.” Research clearly shows that children with more severe pre-operative adenoidal hypertrophy exhibit correspondingly more severe hyponasality (40). Another resonance abnormality is hypernasality, or rhinolalia aperta, where excessive airflow escapes through the nasal cavity when articulating non-nasal vowels and consonants, giving the voice a strong nasal tone. Although hypernasality is uncommon in cases of simple nasal obstruction, it may occur in specific situations, such as in the early post-adenoidectomy period when the velopharyngeal closure mechanism (contact between the soft palate and posterior pharyngeal wall) has not fully adapted to the newly enlarged nasopharyngeal space, potentially leading to temporary hypernasality (40). Additionally, if long-term mouth breathing leads to weakened soft palate muscles due to disuse, it could potentially affect the precision and effectiveness of velopharyngeal closure, thereby creating a predisposition for hypernasality.

Secondly, articulation disorders are often observable. Speech problems are quite prevalent among mouth-breathing children. One systematic assessment indicated that up to 81.7% of mouth-breathing children have speech disorders of varying degrees, with articulation problems being one of the primary clinical manifestations (41). This primarily stems from abnormal tongue posture and specific phoneme errors. Because the tongue is often forced into a low position on the floor of the mouth or protrudes anteriorly during mouth breathing to maintain oral airway patency, this abnormal resting tongue posture and movement pattern directly affect phonemes that require precise elevation of the tongue tip (e.g., against the upper alveolar ridge) or appropriate retraction of the tongue body for accurate production. For example, for alveolar sounds such as /s/, /z/, /t/, /d/, /l/, /n/, mouth-breathing children may exhibit interdentalization of these sounds (e.g., substituting interdental fricatives /*θ*/ and /ð/ for /s/ and /z/, colloquially known as “lisping”) due to the tongue protruding between the teeth, or their pronunciation of these phonemes may be muffled and distorted due to inflexible tongue movement and inaccurate contact points (41). Furthermore, the direct impact of maxillofacial structural abnormalities cannot be ignored. Existing dental malalignments (such as dental crowding, rotations), anterior open bite, and crossbite inherently create physical impediments to the accurate articulation of certain phonemes (especially labiodental and alveolar sounds), making airflow control and coordination of articulatory organs more difficult.

Thirdly, voice disorders may also occur. With mouth breathing, inhaled air bypasses normal nasal warming, humidification, and filtering, and relatively dry, cold air, potentially containing more irritants, directly impacts the pharyngeal and laryngeal mucosa. Over time, this can easily lead to chronic dryness, hyperemia, and inflammation of the pharyngolaryngeal mucosa, thereby affecting the normal physiology of vocal fold vibration and potentially causing voice problems such as hoarseness, rough voice quality, reduced volume, or vocal fatigue (42). It is noteworthy that allergic rhinitis itself is also associated with the occurrence of childhood dysphonia; the two may exacerbate voice problems through common inflammatory mechanisms or mutual influence.

Finally, speech fluency may even be affected, leading to fluency disorders. Preliminary research suggests that the incidence of stuttering (childhood-onset fluency disorder) may also be relatively high among mouth-breathing children (41). The precise underlying mechanisms are not yet fully understood but are hypothesized to involve a combination of factors, including disruption of coordination between breathing and phonation patterns caused by mouth breathing, more effortful phonation, and possibly associated anxiety or social pressure.

## 5 Epidemiological characteristics of “rhinitis face”

### 5.1 Age of onset and high-risk populations

The formation of “rhinitis face” primarily occurs during childhood and adolescence, a critical period of growth and development. During this stage, an individual's maxillofacial skeleton and soft tissues possess significant growth potential and plasticity, rendering them more sensitive to adverse external influences.

Regarding the age distribution of its primary causes, chronic rhinitis (especially allergic rhinitis, AR) and adenoidal hypertrophy (AH) are extremely common conditions in childhood and adolescence. Adenoids, as nasopharyngeal lymphoid tissue, typically reach their peak of physiological enlargement between 2 and 6 years of age. If, during this time, they become excessively enlarged due to recurrent infections, inflammation, or allergic stimuli, leading to airway obstruction, pathological AH develops. Epidemiological data indicate that the prevalence of AH in children can be as high as 49.7% (3). The onset of AR also frequently occurs in childhood, and its course can persist into adulthood, causing long-term distress to patients (18).

The critical window period for facial changes is generally considered to be early, encompassing the preschool and school-age periods (approximately 3–12 years). During this time, persistent nasal obstruction and resultant mouth breathing are crucial drivers for abnormal maxillofacial development. The subjects in the study by (4) were 7–12-year-old children, and its findings suggest this age group represents a key period when facial morphology is significantly affected by mouth breathing. Upon entering adolescence, as individuals are still undergoing rapid growth and development, the continued presence of mouth breathing can still impede the normal length development of the nasomaxillary complex and mandibular growth, as has been demonstrated in animal models (23). Clinical studies also clearly show that dental and skeletal abnormalities associated with mouth breathing are particularly prominent during childhood and adolescence. When individuals reach adulthood and skeletal growth is largely complete, the direct association between mouth breathing and skeletal dysmorphology tends to diminish, with changes more often manifesting as compensatory dental positional adjustments to adapt to the established abnormal skeletal base (26).

Therefore, comprehensively, high-risk populations primarily include children and adolescents suffering from chronic rhinitis (especially moderate-to-severe persistent AR), significant AH, and who consequently exhibit long-term nasal obstruction and habitual mouth breathing. Due to persistent airway obstruction and abnormal breathing patterns, the maxillofacial development of these individuals is most susceptible to adverse effects, leading to the typical “rhinitis face.” Further research indicates that AR is a significant risk factor for persistent obstructive sleep apnea (OSA) in children even after adenotonsillectomy (43), which indirectly highlights the importance of AR in causing long-term upper airway functional problems.

### 5.2 Gender differences

Regarding whether there is a significant gender difference in the incidence of “rhinitis face” itself, direct, large-scale epidemiological research data are relatively scarce. However, indirect clues can be gleaned from the gender distribution of its upstream etiological factors or associated complications.

Firstly, concerning the relationship between mouth breathing and obstructive sleep apnea (OSA), a meta-analysis of OSA-related risk factors in children and adolescents revealed that the overall prevalence of OSA in males is significantly higher than in females (44). Considering the extremely close etiological association between mouth breathing and OSA (mouth breathing being both a common cause and a typical manifestation of OSA), this finding might indirectly suggest that the risk for more pronounced “rhinitis face” changes (especially those involving skeletal structure) associated with severe mouth breathing could be relatively higher in male children.

Secondly, direct evidence exists regarding the gender-specific impact of mouth breathing on children's facial morphology. The study by (4), using 3D facial scanning technology to assess 7–12-year-old children, found that the effect of mouth breathing on facial morphology in this age group indeed exhibited certain gender differences. Specifically, in the 11–12 year age group, male mouth breathers had a significantly greater facial convexity angle than their male nasal-breathing peers. In the 9–10 year age group, female mouth breathers had a greater facial convexity angle than their female nasal-breathing peers, and in the older 11–12 year age group, female mouth breathers had a significantly smaller transverse mandibular width than their female nasal-breathing peers. These findings indicate that the impact of mouth breathing on different facial dimensions may vary between genders and across different age subgroups.

Furthermore, regarding the prevalence of allergic rhinitis (AR), some epidemiological studies show that AR prevalence may be slightly higher in boys than girls during childhood, but in adulthood, female prevalence may surpass that of males, or there may be no significant difference. The specific situation can also be influenced by various factors such as the ethnicity and geographical distribution of the study population. For instance, a survey conducted among AR patients visiting an ENT outpatient clinic showed that the 19–35 age group was the most common age of onset for AR, but this study did not explicitly report gender differences among the overall AR patient cohort (18).

Therefore, based on current information, while a definitive conclusion cannot be drawn regarding a clear gender difference in the overall occurrence of “rhinitis face,” it can be inferred that gender may indeed play a significant modulating role in certain pathogenic stages of its development (such as the risk of OSA) and in specific clinical manifestations (such as changes in facial convexity angle and mandibular width). Future research needs to more directly address the gender distribution and presentation differences of “rhinitis face” itself.

### 5.3 Relevance of different rhinitis types and common allergens

The development of “rhinitis face” is closely associated with various nasal diseases that cause chronic nasal obstruction and mouth breathing, with different types of rhinitis and their specific triggers, especially allergens, playing important roles.

Foremost among these is allergic rhinitis (AR), widely recognized as one of the most common primary diseases leading to “rhinitis face.” The typical clinical symptoms of AR, such as persistent or intermittent nasal obstruction, rhinorrhea, nasal itching, and resultant sneezing, directly form the pathological basis that drives patients (especially children) to develop habitual mouth breathing and perform characteristic behaviors like the “allergic salute” (rubbing the nose upwards). Identification of common allergens is crucial in AR patients. Among specific clinical populations (e.g., patients presenting with nasal symptoms at ENT clinics), dust mites (including *Dermatophagoides pteronyssinus* and *Dermatophagoides farinae*) are frequently reported as one of the main inhalant allergens, with high sensitization rates in many regions worldwide (18). In Northern China, Artemisia pollen is the primary seasonal allergen causing outbreaks of autumn allergic rhinitis, with its sensitization spectrum and pollen dispersal period exhibiting significant regional characteristics (2, 45). Furthermore, allergic reactions triggered by allergenic proteins from pets (such as cats, dogs) found in dander, saliva, or urine are another important cause of AR, and the prevalence of pet sensitization may be gradually increasing among children and adolescents. It is noteworthy that AR patients allergic to cat allergens (more accurately, cat allergenic proteins such as Fel d 1) often tend to have more severe clinical symptoms and a relatively higher risk of comorbid asthma (1).

Secondly, local allergic rhinitis (LAR) is a relatively newer, special clinical phenotype of AR. LAR patients exhibit typical symptoms of allergic rhinitis, but their systemic allergy test results [such as skin prick tests (SPT) or serum specific IgE (sIgE) antibody tests] are usually negative. However, when these patients undergo a nasal provocation test (i.e., direct application of suspected allergens to the nasal mucosa and observation of the local response), a positive allergic reaction can be induced. LAR can also lead to chronic, persistent nasal symptoms such as nasal obstruction and rhinorrhea, thereby contributing to the formation of “rhinitis face,” although its diagnosis is relatively more complex (19).

Furthermore, chronic rhinosinusitis (CRS), whether or not accompanied by nasal polyps [CRS with nasal polyps (CRSwNP) or CRS without nasal polyps (CRSsNP)], can also compel patients to develop habitual mouth breathing and subsequently affect normal facial development if it leads to long-term, severe, and intractable nasal obstruction. The inflammatory mechanisms of CRS are typically complex and may sometimes be associated with allergic factors or a Type 2 (Th2) inflammatory response (an immune response pattern characterized by eosinophilic infiltration and IgE production) (20).

Finally, adenoidal hypertrophy (AH), common in childhood, is another key factor leading to “rhinitis face” (often specifically termed “adenoid facies” in this context). AH frequently coexists with allergic rhinitis or recurrent upper respiratory tract infections and is the most common cause of nasopharyngeal airway obstruction in children. Significantly enlarged adenoids directly obstruct the posterior choanae, leading to severe impairment of nasal ventilation, thereby directly causing persistent mouth breathing and a series of characteristic facial changes (3).

In summary, various nasal diseases can lead to chronic nasal obstruction and, by inducing mouth breathing, contribute to the formation of “rhinitis face.” Among these, allergic rhinitis and associated allergen exposure represent the most common and important links.

## 6 Intervention and management of “rhinitis face”

The intervention for “rhinitis face” is a systematic process requiring multidisciplinary collaboration. Its core principles include early etiological identification, active and effective treatment of the primary disease (critically, relieving nasal airway obstruction), diligent correction of established abnormal breathing patterns and orofacial myofunctional habits, and, when necessary, timely introduction of professional orthodontic treatment or maxillofacial surgical interventions.

### 6.1 Treatment of the primary disease

Effectively controlling or eliminating the primary disease causing nasal obstruction is the initial step in managing “rhinitis face,” aiming to restore or improve normal nasal ventilation function.

1. Pharmacological Treatment is the foundational approach for many types of rhinitis. For allergic rhinitis (AR), first-line therapeutic agents typically include intranasal corticosteroids (INCS). INCS possess potent local anti-inflammatory effects, effectively controlling nasal mucosal inflammation and thereby significantly alleviating core symptoms such as nasal obstruction, rhinorrhea, nasal itching, and sneezing (17, 20). Second-generation oral or nasal antihistamines can rapidly relieve immediate-phase reaction symptoms like sneezing and nasal itching. Leukotriene receptor antagonists (LTRAs), such as montelukast, may serve as an adjunctive treatment option for some AR patients, especially those with comorbid asthma or with nasal obstruction as a primary symptom. Standardized, full-course pharmacological treatment helps to continuously improve nasal ventilation, thereby reducing the frequency and dependence on mouth breathing. For acute or chronic bacterial rhinosinusitis caused by bacterial infection, appropriate antibiotics may be required for a short term under medical guidance, based on the clinical condition and drug sensitivity results. For adenoidal hypertrophy, some studies suggest that topical application of INCS may, to some extent, reduce adenoid volume and improve associated nasal obstruction symptoms, but their overall efficacy is generally less definitive and long-lasting than surgical treatment.
2. Allergen Avoidance and Allergen-Specific Immunotherapy (AIT) are important strategies for intervening in the etiology of AR. For patients diagnosed with AR, after identifying the sensitizing allergens, guidance should be provided to avoid or minimize contact with these allergens in daily life and work. For patients with moderate-to-severe persistent AR who are highly sensitive to major inhalant allergens (such as dust mites, certain pollens) and whose symptoms remain poorly controlled despite standardized pharmacological treatment, AIT [administered via subcutaneous (SCIT) or sublingual (SLIT) routes] is currently the only treatment proven to potentially modify the natural course of the disease and achieve long-term remission. Successful AIT can reduce the patient's sensitivity to specific allergens, leading to long-term symptom improvement and reduced reliance on medication.
3. Surgical Treatment is primarily indicated for patients who have an inadequate response to sufficient pharmacological treatment and have clinically confirmed anatomical abnormalities causing severe nasal obstruction. Common relevant surgeries include adenoidectomy and/or tonsillectomy, which are the primary and effective surgical methods for treating upper airway obstruction and obstructive sleep apnea-hypopnea syndrome (OSAHS) in children caused by excessive adenoidal and tonsillar hypertrophy. Surgery directly relieves physical obstruction in the nasopharynx and oropharynx, thereby effectively expanding the upper airway volume and significantly improving nasal ventilation and nocturnal sleep quality. For persistent nasal obstruction due to severe septal deviation or significant inferior turbinate hypertrophy, septoplasty and/or inferior turbinate reduction surgery may be considered at an appropriate age after the child's growth and development are largely complete (typically recommended after pubertal development is finished to avoid adverse effects on nasofacial growth). A long-term follow-up study on children who underwent a modified “Quick” septoplasty showed that the surgery not only effectively improved their nasal breathing function but might also positively influence subsequent dentofacial morphology, promoting the harmonious development of craniofacial structures and dental occlusion (46).

### 6.2 Correction of poor breathing habits and orofacial myofunctional therapy (OMT)

After significantly improving or substantially restoring normal nasal ventilation through pharmacological treatment or surgical intervention, it is crucial to actively guide patients (especially children) to re-establish and consolidate correct nasal breathing patterns. However, some patients may involuntarily maintain mouth breathing due to long-established habits even after their nasal passages are patent. In such cases, orofacial myofunctional therapy (OMT), a non-invasive behavioral intervention method, can play an important role. OMT is a comprehensive therapeutic system comprising specific exercises and practices aimed at correcting abnormal orofacial muscle functions, such as habitual mouth breathing, abnormal swallowing patterns (e.g., tongue thrust swallowing), incorrect tongue resting posture, and lip incompetence.

1. The core goals of OMT are to help patients re-establish a physiological, nasal-dominant breathing pattern, while simultaneously improving the normal posture and function of lip and tongue muscles (e.g., the tongue should rest lightly against the palate, and lips should be naturally sealed), and ultimately promoting coordinated movement and functional balance of the entire perioral musculature.
2. The specific content of OMT is typically individualized based on the patient's particular issues but may include the following aspects: training for lip seal competence (e.g., practicing tight lip closure, using specialized lip tapes at night to assist mouth closure), tongue muscle function training (e.g., practicing precise tongue tip placement on a specific palatal spot like the “N-spot,” performing tongue clicks, tongue suction against the palate to strengthen tongue muscles and improve tongue position), re-education and training of breathing patterns (e.g., teaching diaphragmatic breathing, conscious slow deep nasal breathing exercises with a closed mouth), and necessary chewing and swallowing function training (to correct abnormal compensatory swallowing movements).
3. Regarding the clinical efficacy of OMT, a scoping review on OMT for improving orofacial functions and habits indicated that although many preliminary clinical studies have reported positive therapeutic effects of OMT in improving mouth breathing status and promoting natural lip closure, there is currently insufficient high-quality evidence-based medicine [e.g., large-scale randomized controlled trials (RCTs), long-term follow-up data on treatment efficacy] to fully and definitively confirm its precise efficacy in all clinical situations, optimal training protocols, and the long-term stability of its effects (47). Therefore, the application of OMT currently still emphasizes an individualized approach and may need to be closely integrated with other therapeutic methods (such as orthodontic treatment) to achieve optimal results. However, there is a consensus that early diagnosis and timely intervention for mouth breathing behavior can help interrupt or correct established abnormal neuromuscular reflex patterns, thereby avoiding their persistent detrimental impact on the normal maxillofacial growth and development of affected children (5).

### 6.3 Timely intervention of orthodontic and maxillofacial surgical treatment

For patients who have already developed relatively obvious maxillofacial skeletal deformities and dental malocclusions due to long-term mouth breathing or other reasons, a comprehensive assessment by a specialist orthodontist is typically required after their primary disease (e.g., rhinitis, adenoidal hypertrophy) has been effectively controlled and their nasal breathing pattern is substantially established or significantly improved. A corresponding orthodontic treatment plan is then formulated and implemented based on the specific situation (27).

1. Early Orthodontic Treatment (also known as growth modification or interceptive orthodontics) primarily targets children still in their peak growth and development period. If clinical examination and imaging assessment reveal maxillary transverse deficiency in a child (manifesting as a narrow dental arch, posterior crossbite, etc.), rapid maxillary expansion (RME) is a commonly used and proven effective early intervention method. By applying specific transverse expansion forces before the child's midpalatal suture has completely ossified, RME can effectively open the suture, thereby significantly increasing the transverse width of the maxilla and expanding the width and volume of the nasal cavity. This series of anatomical changes can directly reduce nasal airflow resistance, improve nasal ventilation, and create favorable conditions for mouth-breathing patients to transition to a normal nasal breathing pattern (28, 48–51). Furthermore, RME has been shown to have a positive effect on improving symptoms related to pediatric obstructive sleep apnea syndrome (OSAS) and reducing the apnea-hypopnea index (AHI) (48). An interesting study even found that after RME treatment, the volume of adenoids and tonsils might also significantly decrease in some children, potentially offering a new mechanistic explanation for how RME improves upper airway patency and suggesting its effects may not be limited to the nasal cavity and maxilla (52). The long-term effects of RME treatment have also received attention, with multiple studies showing that the improvements in nasal structure and function can remain relatively stable after treatment completion (30, 49). For adolescent or young adult patients with higher skeletal maturity (e.g., midpalatal suture has begun or largely completed osseous fusion), surgically assisted rapid maxillary expansion (SARME) may be required if transverse maxillary expansion is still needed (53).
2. Comprehensive Orthodontic Treatment is primarily applicable to patients in late stages of growth and development or those who have reached adulthood. For established malocclusions, such as dental crowding, maxillary incisor protrusion, anterior open bite, or deep overbite, comprehensive tooth movement and occlusal adjustment are usually achieved through the use of conventional fixed appliances (colloquially “braces”) or, increasingly, clear aligners (such as transparent vacuum-formed aligners) to achieve ideal dental alignment, aesthetics, and function.
3. Orthognathic Surgery is mainly indicated for adult patients (whose growth and development have generally ceased) with severe skeletal discrepancies (e.g., severe mandibular retrognathia or prognathism, maxillary hypoplasia or hyperplasia, significant facial asymmetry) where orthodontic treatment alone cannot resolve the fundamental skeletal problem. Such patients often require a combined treatment approach involving orthodontic treatment and orthognathic surgery. Surgical procedures precisely alter the position and morphology of the jaws, complemented by pre- and post-surgical orthodontic treatment to fine-tune dental occlusion, ultimately achieving ideal facial improvement and occlusal function reconstruction.
4. Regarding considerations for early orthodontic treatment, it must be emphasized, as noted by (27), that the success of early orthodontic treatment is highly dependent on accurate identification of indications and correct timing of treatment. Not all children presenting with mouth breathing or so-called “adenoid facies” can have all their problems completely resolved by early orthodontic treatment alone. In many cases, multidisciplinary collaboration (including otolaryngology, orthodontics, sleep medicine, and even allergology) is crucial for developing comprehensive, individualized treatment plans.

### 6.4 Health education for patients and families

Providing thorough, detailed, and easily understandable health education to the patient and their parents (or guardians) is an indispensable key component in the successful management of “rhinitis face.” The goals of health education are to enhance their understanding of the condition, improve treatment adherence, and promote positive lifestyle changes. Specific content should include:

1. Clearly explaining the potential harms of chronic rhinitis and long-term mouth breathing. Parents and children with sufficient comprehension should understand that these are not merely “minor ailments” but can have profound adverse effects on maxillofacial growth and development, nocturnal sleep quality, and even long-term overall quality of life (including learning, social interactions, and psychological health).
2. Emphasizing the importance of early diagnosis and standardized treatment of the primary disease. Parents should be informed that for common primary diseases causing nasal obstruction, such as allergic rhinitis (AR) and adenoidal hypertrophy (AH), the earlier an accurate diagnosis is made and standardized, continuous treatment (whether pharmacological, immunological, or, if necessary, surgical) is initiated, the more effectively the condition can be controlled and nasal ventilation improved, thereby maximally reducing or preventing the occurrence and progression of “rhinitis face.”
3. Providing detailed guidance on the necessity and specific methods for establishing and maintaining good nasal breathing habits. After nasal ventilation issues are resolved or improved, active guidance is needed to help the child consciously resume and persist in using nasal breathing. Simple, feasible methods can be taught, such as closed-mouth puffing exercises, self-reminders to breathe through the nose during waking hours, and targeted orofacial myofunctional therapy (OMT) under the guidance of a doctor or therapist.
4. Stressing the critical role of adherence to OMT or orthodontic treatment for final efficacy. Both orofacial myofunctional therapy and orthodontic correction typically require long-term, active cooperation and persistence from the patient (and parents). They need to understand that treatment success depends not only on the clinician's skill but also significantly on factors such as attending appointments on time, diligently completing home exercises, and correctly wearing and maintaining appliances. Good adherence is a vital guarantee for achieving desired treatment outcomes.
5. Clarifying the significance of regular follow-up visits. Parents and patients should be informed that even after the current treatment phase is completed, regular follow-up visits are necessary as per medical advice. This helps monitor the stability of the condition, assess the durability of treatment effects, promptly detect and manage any new problems or signs of recurrence, and adjust subsequent management strategies according to the child's growth and developmental changes.

## 7 Discussion on “aesthetic preference for rhinitis face”

When discussing the various aspects of “rhinitis face,” a noteworthy point is the so-called notion that “people like girls with a rhinitis face.” In this systematic review of medical literature, no direct evidence based on scientific research data was found to support a universal aesthetic preference for “rhinitis face.” The medical field's focus on “rhinitis face” (or “adenoid facies”) has consistently and primarily centered on its clear pathophysiological mechanisms, its diverse negative impacts on patient health (especially in children and adolescents, including maxillofacial development, respiratory function, sleep quality, and psychological health), and methods for accurate clinical diagnosis and effective multidisciplinary treatment and management.

Any perceived “aesthetic preference” for certain features of “rhinitis face,” if it indeed exists in specific populations or subcultures, is more likely a highly subjective personal feeling, reflecting the diversity of individual aesthetic standards, or a short-term influence of online cultural phenomena, social media trends, and aesthetic imagery shaped by certain artistic works within specific historical periods or regional cultural contexts. Such phenomena lack a universal, cross-cultural scientific basis and robust medical consensus for support.

From a purely medical and health science perspective, many core features encompassed by “rhinitis face,” such as persistent open-mouth posture, dark circles under the eyes due to venous stasis, relative midface hypoplasia, mandibular retrognathia, and various dental malalignments and malocclusions, are not healthy physiological manifestations. On the contrary, they are pathological changes directly related to potential health impairments or compensatory manifestations of the body adapting to pathological states (such as chronic nasal obstruction). These features are clinical health warning signs that require active identification, assessment, and intervention, rather than aesthetic traits to be socially pursued, admired, or romanticized. Clearly and accurately conveying this medical viewpoint to the public is of crucial public health significance and educational value in guiding society to correctly understand the potential health risks associated with “rhinitis face” and promoting early detection and scientific intervention.

## 8 Conclusion and outlook

This review, centered on the clinical phenomenon of “rhinitis face” (or “adenoid facies”), has systematically addressed several core questions posed in the introduction. Firstly, it has clearly defined its clinical concept and comprehensively depicted its diverse clinical manifestations, including persistent open-mouth posture, characteristic midface hypoplasia with excessive vertical growth (forming a “long face type”), mandibular retrognathia, narrow dental arches with various malocclusions, and a series of periorbital (e.g., allergic shiners, Dennie-Morgan lines, eyelash trichomegaly) and nasal (e.g., transverse nasal crease) skin changes. Secondly, it has delved into its core pathophysiological mechanisms, emphasizing that mouth breathing patterns triggered by chronic nasal airway obstruction are the initiating factor. This pattern, by disrupting orofacial muscle balance, interfering with normal tongue function, and affecting the maxillofacial skeletal growth trajectory, in conjunction with local inflammation and mechanical irritation, collectively contributes to the formation of these complex facial characteristics. Furthermore, this review has systematically summarized the various objective clinical methods and ancillary diagnostic tools used to assess “rhinitis face,” covering multiple dimensions from detailed history taking and physical examination to imaging techniques (x-ray, CT, CBCT), cephalometric analysis, nasal patency tests, orofacial myofunctional assessment, and allergen testing. Crucially, this paper has comprehensively elucidated the non-negligible long-term impacts and potential complications of “rhinitis face.” These impacts are not limited to aesthetics but extend to severe maxillofacial skeletal deformities, dental malocclusions, temporomandibular joint dysfunction, sleep-disordered breathing, and may profoundly adversely affect an individual's respiratory physiology, exercise tolerance, speech clarity, and even psychological health and overall quality of life. Finally, this review has outlined and evaluated current multidisciplinary intervention strategies and comprehensive management plans for “rhinitis face,” stressing the importance of early recognition, active treatment of the primary nasal disease, correction of poor mouth breathing habits, and, when necessary, integration of orthodontic or surgical treatment.

In summary, “rhinitis face” encompasses a series of characteristic changes in children and adolescents resulting from chronic nasal diseases and secondary mouth breathing. Its developmental mechanisms are complex, and its clinical impact is far-reaching. Effective intervention and management rely on multidisciplinary collaboration and early intervention. Future research should aim to further elucidate the more detailed pathogenesis and cellular and molecular biological basis behind various facial features (especially phenomena like “long eyelashes” and “large eyes”), and actively explore and validate more sensitive and specific early diagnostic biomarkers or non-invasive assessment tools. Simultaneously, there is a need to continuously optimize existing intervention measures (especially orofacial myofunctional therapy and early orthodontic treatment) regarding the selection of indications, optimal timing of treatment, standardized operational protocols, and evidence-based assessment systems for long-term efficacy. Additionally, strengthening attention to, systematic assessment of, and effective support for the potential psychosocial impacts on patients with “rhinitis face” is an important aspect not to be overlooked in future work. Regarding the precise association between certain facial features (“long eyelashes, large eyes”) and rhinitis, and their so-called “aesthetic preference,” the former requires more rigorous scientific research to unveil its intrinsic connections, while the latter clearly lacks scientific basis. Clinical practice should always be guided by evidence-based medicine and an objective, scientific health perspective, providing correct popular science communication and health education to the public, emphasizing that “rhinitis face” is primarily a health issue requiring medical attention and scientific management, and not otherwise.
